# Disease Severity Indexes and Treatment Evaluation Criteria in Vitiligo

**DOI:** 10.1155/2011/750342

**Published:** 2011-05-22

**Authors:** Tamihiro Kawakami, Takashi Hashimoto

**Affiliations:** ^1^Department of Dermatology, St. Marianna University School of Medicine, 2-16-1 Sugao, Miyamae-ku, Kawasaki, Kanagawa 216-8511, Japan; ^2^Department of Dermatology, Kurume University School of Medicine and Institute of Cutaneous Cell Biology, 67 Asahimachi, Fukuoka 830-0011, Japan

## Abstract

There is a current lack of consensus regarding methods of assessment of vitiligo. Recently, the Vitiligo Area Scoring Index (VASI) and the Vitiligo European Task Force (VETF) were proposed to offer more accurate measures of disease severity indexes and treatment evaluation criteria. It would make sense to combine the VASI with the VETF system. We proposed an original scale for treatment evaluation criteria in vitiligo based on VASI. We plan to add the digital image analysis system, health-related quality of life questionnaire, affected skin location, and skin color in the original scale.

## 1. Introduction

Vitiligo, the most common depigmenting disorder, affects 0.5–1% of the worldwide population, causing disfigurement and serious disturbances in quality of life. There is a current lack of consensus on methods of assessment of this disorder, which makes it generally impossible to perform meta-analyses or compare the outcomes of different studies of the same treatment or parameter. Recently, the Vitiligo Area Scoring Index (VASI) [1] and the Vitiligo European Task Force (VETF) [2] tools were proposed to offer more accurate measures of disease severity indexes and treatment evaluation criteria compared to simple clinical photography alone. VASI provides a relatively simple method, analogous to the Psoriasis Area Severity Index (PASI), to measure repigmentation. Using tools developed by VETF vitiligo and treatment outcomes can be assessed using a system that combines analysis of extent and stage of disease and disease progression [3]. Extent is evaluated by the rule of nines, staging is based on cutaneous and hair pigmentation, and spreading is assessed based on Wood's light examination. 

## 2. Vitiligo Area Scoring Index (VASI)

Hamzavi et al. [1] have introduced a quantitative parametric score, named VASI for Vitiligo Area Scoring Index, which is conceptually derived from the PASI score widely used in psoriasis assessment [3]. The total body VASI is calculated using a formula that includes contributions from all body regions (possible range, 0–100)

(1) $$\begin{array}{cc} & \text{VASI}\\ & =\underset{\text{All Body Sites}}{\sum }\left[\text{Hand Units}\right]\times \left[\text{Residual Depigmentation}\right]. \end{array}$$

One hand unit, which encompasses the palm plus the volar surface of all the digits, is approximately 1% of the total body surface area [4] and is used as a guide to estimate the baseline percentage of vitiligo involvement in each body region. The body is divided into five separate and mutually exclusive regions: hands, upper extremities (excluding hands), trunk, lower extremities (excluding feet), and feet. The axillary region is included with the upper extremities while the buttocks and inguinal areas are included with the lower extremities. The extent of residual depigmentation is expressed by the following percentages: 0, 10%, 25%, 50%, 75%, 90%, or 100%. At 100% depigmentation, no pigment is present; at 90%, specks of pigment are present; at 75%, the depigmented area exceeds the pigmented area; at 50%, the depigmented and pigmented areas are equal; at 25%, the pigmented area exceeds the depigmented area; at 10%, only specks of depigmentation are present.

## 3. Vitiligo European Task Force (VETF)

The VETF proposed a system that combines analysis of extent, stage of disease (staging), and disease progression (spreading) [2]. Extent is evaluated using the rule of nines [5], already used in atopic dermatitis assessment [6]. Staging is based on cutaneous and hair pigmentation in vitiligo patches, and the disease is staged 0–3 on the largest macule in each body region, except hands and feet, which are assessed separately and globally as one unique area. 

A proposal was made for simplifying the staging scale:

stage 0: normal pigmentation (no depigmentation in area graded),
stage 1: incomplete depigmentation (including spotty depigmentation, trichrome, and homogeneous lighter pigmentation)
stage 2: complete depigmentation (may include hair whitening in a minority of hairs, <30%),
stage 3: complete depigmentation plus significant hair whitening (>30%).

“Spreading” in VETF was introduced to include a dynamic dimension, since rapidly progressive vitiligo needs urgent intervention to stabilize the disease. The proposed grid allows scoring this dimension on a simple scale (+1: progressive; 0: stable; −1: regressive). Spreading is assessed by combining Wood's lamp and electric light examinations in a dark room. Wood's lamp includes a magnifying lens to assess hairs, especially vellus hairs.

## 4. Discussion

It is difficult to compare the efficacy of different treatment modalities in vitiligo [7]. Based on the currently available literature, it would make sense to combine the depigmentation scale of VASI with the VETF system. The VETF system cannot always be easily handled in clinical practice because Wood's lamp examination is troublesome. In addition, Wood's lamp may not be popular in non-European countries. The improvement of vitiligo for therapy is more slowly compared to that of psoriasis in general. We suggest that a 50% reduction in VASI score (VASI 50) equates a clinical improvement of a 75% reduction in PASI score (PASI 75). Therefore, we propose an original scale for treatment evaluation criteria in vitiligo as follows: very much improved (increased more than 50 points of VASI score), much improved (25–50 points increase of VASI score), improved (10–25 points increase of VASI score), minimally improved (less than 10 points increase of VASI score). In contrast, very much worse (decreased more than 50 points of VASI score), much worse (25–50 points decrease of VASI score), worse (10–25 points decrease of VASI score), and minimally worse (less than 10 points decrease of VASI score) (Table 1). A new digital image analysis system (DIAS) for the surface measurement of vitiligo lesions was proposed [8]. Extending the system to include 3D information would probably allow much larger areas to be measured reliably. We may adopt the DIAS for treatment evaluation in vitiligo.

An important aspect of the therapeutic response is how the patients feel about their vitiligo after the treatment because there is a psychological point of view to the disease. Vitiligo is often immediately visible to others, and those with the condition may suffer social and emotional consequences including low self-esteem, social anxiety, depression, stigmatization, and, in extreme cases, rejection by those around them [9]. Assessment of treatment efficacy should include quality of life with regard to the psychological effects. One way of evaluating the burden of vitiligo is by measuring the health-related quality of life (HRQL) using standardized questionnaires to measure the impact of a disease on the physical, psychologic, and social functioning, and the well-being of the patient [5]. We will add dermatology-specific HRQL questionnaire such as Skindex and Dermatology Life Quality Index in our original scale.

The Pemphigus Disease Area Index (PDAI) has twofold higher score for oral involvement compared to skin involvement [10]. Depigmentation on the face and neck seems to be psychologically more harmful than that on trunk and extremities. Severity Indexes of vitiligo on the face and neck should receive twofold higher scoring than that on the rest of the body in analogy with the PDAI. In addition, skin color (skin types I to VI according to Fitzpatrick) should be assessed separately in the disease severity indexes and treatment evaluation criteria. In people with a pale white skin color (skin type I–III), vitiligo may cause little concern. In contrast, yellow and black people with dark skin (skin type IV–VI) may be anxious about depigmentation. Some additional clinical studies would be required to adequately address affected skin location and skin color in disease severity indexes and treatment evaluation criteria of vitiligo.

## Figures and Tables

**Table 1:** 

VASI score	~−50	Very much worse
VASI score	−50 ~ −25	Much worse
VASI score	−25 ~ −10	Worse
VASI score	−10 ~ 0	Minimally worse
VASI score	0 ~ +10	Minimally improved
VASI score	+10 ~ 25	Improved
VASI score	+25 ~ 50	Much improved
VASI score	+50~	Very much improved
